# An Additive Effect of Promoting Thermogenic Gene Expression in Mice Adipose-Derived Stromal Vascular Cells by Combination of Rosiglitazone and CL316,243

**DOI:** 10.3390/ijms18051002

**Published:** 2017-05-08

**Authors:** You-Lei Li, Xiao Li, Tian-Tuan Jiang, Jia-Min Fan, Xue-Li Zheng, Xin-E Shi, Tai-Yong Yu, Gui-Yan Chu, Gong-She Yang

**Affiliations:** 1 Laboratory of Animal Fat Deposition and Muscle Development, College of Animal Science and Technology, Northwest A&F University, Yangling 712100, Shaanxi, China; liyoulei@nwsuaf.edu.cn (Y.-L.L.); nicelixiao@nwsuaf.edu.cn (X.L.); jiangtt@gsau.edu.cn (T.-T.J); zhengxueli@nwsuaf.edu.cn (X.-L.Z.); xineshi@163.com (X.-E.S.); yutaiyong310@nwsuaf.edu.cn (T.-Y.Y.); yz97721@gmail.com (G.-Y.C.); 2 College of Animal Science and Technology, Gansu Agriculture University, Lanzhou 730070, Gansu, China; 3 College of Life Sciences, Northwest A&F University, Yangling 712100, Shaanxi, China; 18821714887@163.com

**Keywords:** adipocyte browning, rosiglitazone, CL316,243, beige, brite, thermogenic gene, additive effect

## Abstract

It is well-documented that CL316,243 (a β3 agonist) or rosiglitazone (a PPARγ agonist) can induce white adipocyte populations to brown-like adipocytes, thus increasing energy consumption and combating obesity. However, whether there is a combined effect remains unknown. In the present study, stromal vascular cells of inguinal white adipose tissue (iWAT-SVCs for short) from mice were cultured and induced into browning by CL316,243, rosiglitazone, or both. Results showed that a combination of CL316,243 and rosiglitazone significantly upregulated the expression of the core thermogenic gene *Ucp1* as well as genes related with mitochondrial function (*Cidea*, *Cox5b*, *Cox7a1*, *Cox8b*, and *Cycs*), compared with the treatment of CL316,243 or rosiglitazone alone. Moreover, co-treatment with rosiglitazone could reverse the downregulation of *Adiponectin* resulting from CL316,243 stimuli alone. Taken together, a combination of rosiglitazone and CL316,243 can produce an additive effect of promoting thermogenic gene expression and an improvement of insulin sensitivity in mouse iWAT-SVCs.

## 1. Introduction

Excessive white adipose tissue accumulation in overweight and obese individuals is the major factor for developing a series of metabolic diseases, such as insulin resistance, type 2 diabetes, cardiovascular disease, and certain cancers [1]. Brown adipocyte can dissipate chemical energy (fatty acid and glucose) to produce heat and are important in regulating body temperature and body weight [2,3]. Recently, brown-like adipocytes, so-called beige/brite adipocytes, were identified in white adipose tissue [4,5]. Beige or brite adipocytes, like classical brown adipocytes, express relatively high UCP1 protein to convert energy into heat [6]. Therefore, enhancing white fat “browning” would be helpful to fight against obesity and associated disease.

Research had shown that rosiglitazone and CL316,243 could effectively promote browning of white fat and protect mice from diet-induced obesity [7,8]. Rosiglitazone, a synthetic PPARγ agonist, can induce brown-specific gene expression in white adipocytes in vitro and in vivo [4,7]. Generally, the brown-like adipocytes recruited by exposure to PPARγ ligands are often referred to as brite adipocytes [9,10]. Besides, β3-agonist CL316,423 or cold stimuli can also induce the brown-like cells in WAT (white adipose tissue) by activating β3-adrenoceptor pathway, and this kind of brown-like cell is referred to as beige adipocyte [10]. Thus, it remains an open question whether a combination of rosiglitazone and CL316,243 can increase the effect of browning.

Compared with eWAT (epididymal white adipose tissue), iWAT (inguinal white adipose tissue) are more prone to induce browning phenotype, including multilocular lipid droplets, higher *Ucp1* expression and densely packed mitochondrion [11,12,13]. Recent studies have shown that CL316,243-induced beige adipocytes are distinct from rosiglitazone-induced brite adipocytes in gene expression patterns and origins [6,10]. Despite having those unclarified differences, both beige and brite adipocyte progenitors are presented in iWAT-SVCs, so iWAT-SVCs is a reliable cellular system to study the development of beige or brite cells in vitro [14].

In our study, iWAT-SVCs were used to evaluate the effect of the rosiglitazone, CL316,243, or both on the thermogenesis. Our results indicated an additive effect of promoting thermogenic gene expression and mitochondrial biogenesis by a combination of rosiglitazone and CL316,243 in mice iWAT-SVCs. This finding might provide a potential therapeutic strategy to fight against obesity or related diseases.

## 2. Results

### 2.1. Lacked Thermogenic Characteristics Under Standard White Adipogenic Conditions

**Figure 1:**
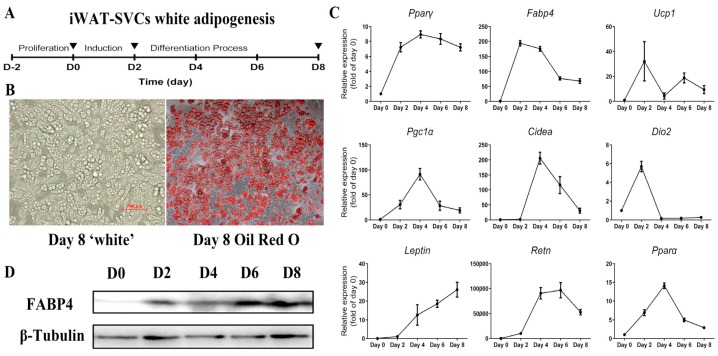
Gene expression profiles during mouse iWAT-SVC white adipogenesis. (**A**) Protocol used to differentiate iWAT-SVCs into white adipocytes (Day 0: add induction medium; Day 2: change medium to maintenance medium for 6 days, and change medium every 2 days; Day 8: harvest cells for Oil Red O staining); (**B**) Fully differentiated adipocytes at Day 8 and Oil Red O staining of cells differentiated at Day 8, respectively; (**C**) qPCR analysis of adipogenic genes (*Pparγ* and *Fabp4*), brown-selective genes (*Ucp1*, *Pgc1α*, *Cidea*, and *Dio2*) and white-selective genes (*Leptin* and *Resistin*); (**D**) Western blot analyses of adipocyte marker FABP4 in differentiated cells. * *p* < 0.05, ** *p* < 0.01. qPCR data were normalized to Day 0 and represented as Mean ± SEM.

To investigate the effect of rosiglitazone and CL316,243 in fat browning, we isolated and induced iWAT-SVCs to white adipocyte as Figure 1A, then detected the basal expression level of thermogenic genes. We harvested the cultured cells every two days until fully differentiation (Day 8, as shown in Figure 1B). During iWAT-SVC white adipogenesis, transcription levels of common adipogenic genes (*Pparγ* and *Fabp4*) showed an increase at Day 2 or Day 4 but soon slowly dropped down (Figure 1C). Notably, brown-specific genes (*Ucp1*, *Pgc1α*, and *Cidea*) were expressed at relatively low levels with some fluctuations (Figure 1C). There was a ~25 folds’ increase (*Leptin*) and ~100,000 folds’ increase (*Resistin*) during white adipogenesis, respectively (Figure 1C). Western blot analysis of adipogenic marker FABP4 also showed an increase during differentiation (Figure 1D), while the expression of UCP1 protein was almost undetectable (not shown). These data confirmed that this cellular system can be used to explore the effects of rosiglitazone and CL316,243 in browning.

### 2.2. CL316,243 Induced the Cultured Mature White Adipocyte Beiging

**Figure 2:**
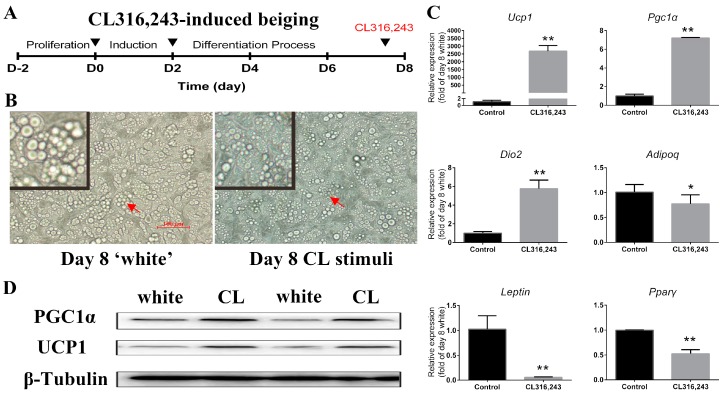
CL316,243 induced iWAT-SVC-derived mature white adipocyte beiging. (**A**) Cultured mature white adipocytes were treated with CL316,243 (Day 0: add induction medium; Day 2: change medium to maintenance medium; 4 hours before day 8: add CL316,243); (**B**) The lipid droplets turn smaller after CL316,243 treatment. Red arrow part was amplified on the upper left area; (**C**) qPCR analysis of expressions of brown-selective genes (*Ucp1*, *Pgc1α*, *Dio2*, and *Pparα*), white-selective genes (*Leptin*), and adipogenic genes (*Adiponectin* and *Pparγ*); (**D**) Western blot analyses of thermogenic markers UCP1 and PGC1α in differentiated cells. * *p* < 0.05, ** *p* < 0.01. Data was represented as Mean ± SEM.

Next, we conducted the experiment on cultured mature white adipocytes to induce browning by CL316,243 treatment for 4 h (Figure 2A). This system allowed us to efficiently transform mature white adipocytes into beige adipocytes through adrenergic activation [15]. Compared with mature white adipocytes at Day 8, the lipid droplets turned smaller when treated with CL316,243 (Figure 2B). Brown-specific genes (*Ucp1*, *Pgc1α*, and *Dio2*) increased significantly (Figure 2C). However, the white adipocyte selective gene *Leptin* and the master adipogenic gene *Pparγ* show a dramatic decrease (Figure 2C). Western blot analysis suggested that the expression of UCP1 and PGC1α, the core thermogenic genes, were induced rapidly by CL316,243, which was consistent with the qPCR results (Figure 2D). Unexpectedly, CL316,243 treatment had a significant impairment of *Adiponectin* expression (Figure 2C). These expression profiles of mature white adipocytes in response to CL316,243 further reinforced the notion that CL316,243 can effectively induce browning of iWAT-SVCs.

### 2.3. Differentiation of Mice iWAT-SVCs to Brite Cells in the Presence of Rosiglitazone

**Figure 3:**
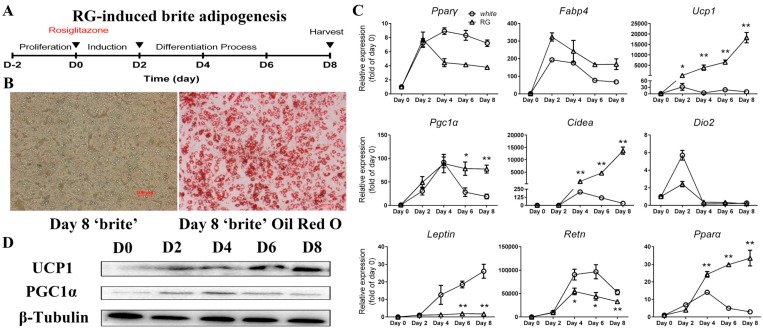
Expression profiles of mice iWAT-SVC brite adipogenesis. (**A**) Protocol used to induce iWAT-SVCs into brite adipocytes (Day 0: add induction medium containing rosiglitazone; Day 2: change medium to maintenance medium with rosiglitazone; Day 8: harvest cells for Oil Red O staining); (**B**) Fully differentiated brite adipocytes at Day 8 and Oil Red O staining of brite cells differentiated at Day 8, respectively; (**C**) qPCR analysis of adipogenic genes, brown-selective genes, and white-selective genes; (**D**) Western blot analyses of thermogenic markers UCP1 and PGC1α during brite differentiation. * *p* < 0.05, ** *p* < 0.01. Data was represented as Mean ± SEM.

Here, we used another protocol for inducing iWAT-SVCs to brite cells by PPARγ agonist rosiglitazone (Figure 3A) [14]. Morphological observation showed that adipocyte cells had completely differentiated with more multilocular lipid droplets (Figure 3B). During iWAT-SVC brite adipogenesis, similar to iWAT-SVC white adipogenesis, expression levels of common adipogenic genes (*Pparγ* and *Fabp4*) showed a significant increase at Day 2 but slowly dropped afterward (Figure 3C). Consistent with other studies, brown-specific genes (*Ucp1*, *Pgc1α*, and *Cidea*) showed a sharp increase during brite adipogenesis, especially *Ucp1* and *Cidea*, about 18,000-fold and 13,000-fold higher at Day 8 vs. Day 0, respectively (Figure 3C). However, the expression level of *Dio2*, a marker of white adipocyte browning, did not have a huge difference between white adipogenesis and brite adipogenesis (Figure 3C). We next detected two white adipocytes selective genes (*Leptin* and *Resistin*). We found a no more than two-fold increase of *Leptin* and a 55,000-fold increase of *Resistin* during brite adipogenesis, respectively (Figure 3C). Both *Leptin* and *Resistin* were inhibited in brite adipogenesis compared with white adipogenesis, especially *Resistin*. Western blot analysis of thermogenic markers UCP1 and PGC1α also showed an increase during differentiation (Figure 3D), which was consistent with the qPCR results.

### 2.4. CL316,243 Further Promoted Thermogenic Gene Expression and Mitochondrial Biogenesis of Rosiglitazone-Induced Brite Adipocytes

**Figure 4:**
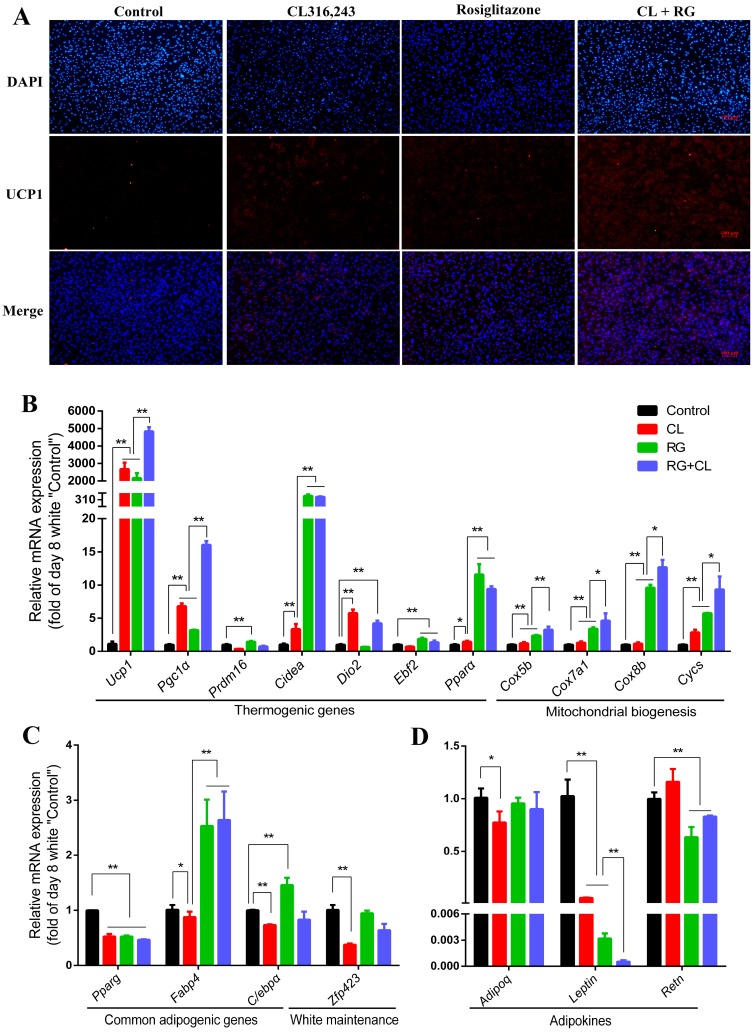
CL316,243 and rosiglitazone additively induced thermogenic gene expression and mitochondrial biogenesis. (**A**) UCP1 immunostaining in Control and CL/RG/CL + RG induced brown-like adipocytes. Fluorescence color: blue (nuclei), red (UCP1). Scale bar, 100 µm; (**B**) qPCR analysis of thermogenic genes, mitochondrial function genes; (**C**) Level of common adipogenic genes, white adipocytes maintenance gene; (**D**) Adipokines mRNA expression in Control (Day 8 white adipocyte), CL (CL316,243 treatment), RG (rosiglitazone treatment), and RG + CL (rosiglitazone followed by CL316,243 treatment). The data showed the fold changes of the expression for the target genes at Day 8. Data was represented as mean ± SEM; *p*-values were calculated by one-way ANOVA (* *p* < 0.05, ** *p* < 0.01).

Next, iWAT-SVCs were treated with rosiglitazone for 8 days during differentiation and followed by CL316,243 for 4 h before harvest (RG + CL group). We observed comparative levels of *Ucp1* gene between the CL and RG groups. Intriguingly, *Ucp1* expression of the RG + CL group was significantly higher than the RG or CL group, suggesting CL316,243 could further promote *Ucp1* expression of rosiglitazone-induced brite adipocytes (Figure 4A,B). More strikingly, treatment with rosiglitazone followed by CL316,243 further promote mitochondrial biogenesis. Mitochondrial function genes such as *Cidea*, *Cox5b*, *Cox7a1*, *Cox8b*, and *Cycs*, had a significant rise in RG + CL group compared with the RG or CL group (Figure 4B). Besides, the expressions of *Resistin* and *Leptin* were significantly inhibited in the RG + CL group compared with the control group, while the *Adiponectin* expression was not altered (Figure 4D).

**Figure 5:**
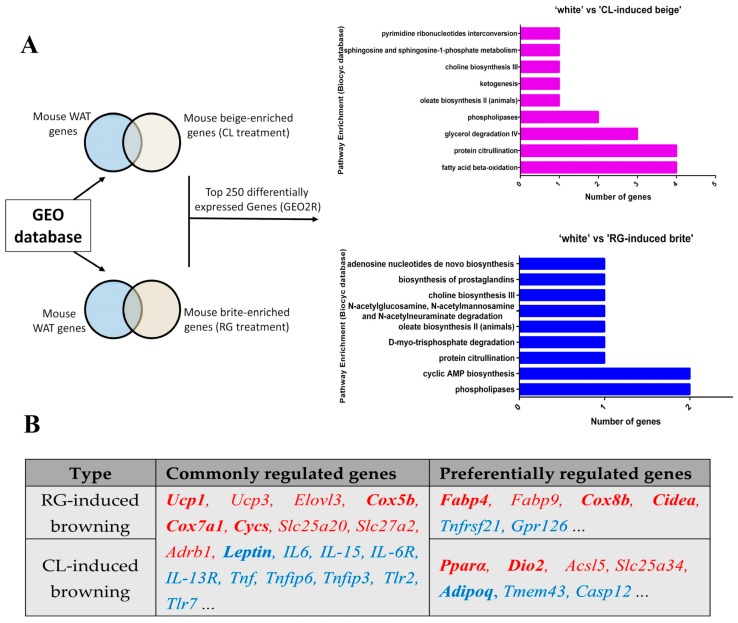
The unique regulatory roles of CL316,243 and rosiglitazone in adipocyte browning process. (**A**) CL316,243 and rosiglitazone preferentially activates different metabolic pathways involved in adipocyte browning (Common pathways: phospholipase, protein citrullination, and oleate/choline biosynthesis; Preferential pathways: CL-induced fatty acid β-oxidation, RG-induced cAMP biosynthesis); (**B**) Commonly and preferentially regulated genes of RG-induced browning and CL-induced browning from dataset GSE87,191 (gene names in red represent upregulating genes, while those in blue represent downregulating genes, and gene names in bold have been checked in our study).

Finally, to make it clear why rosiglitazone and CL316,243 have a combined effect during browning, we used bioinformatic tools to identify genes that were differentially expressed in microarray data (GSE87,191: the mRNA signature of white adipocytes, rosiglitazone-induced brite adipocytes, and CL-induced beige adipocytes), which was obtained from the GEO (Gene Expression Omnibus) database. Further, we used the Biocyc database to do pathway enrichment analysis for these differentially expressed genes (listed in Table S2). As illustrated in Figure 5A, rosiglitazone and CL316,243 have common and unique regulatory roles in adipocyte browning process. They both changed the phospholipase, protein citrullination, and oleate/choline biosynthesis. It is remarkable that CL316,243 preferentially induced fatty acid β-oxidation, while rosiglitazone induced cAMP biosynthesis. In addition, parts of CL- and RG-regulated genes were listed in Figure 5B, and genes in bold (*Ucp1*, *Cox5b*, *Cox7a1*, and *Cycs*) were consistent with the finding in our study. These data suggest CL and RG have similar (such as promoting thermogenesis) and complementary (such as mitochondrial biogenesis and insulin sensitivity regulation) role in its function and effect.

## 3. Discussion

In our study, either CL316,243 or rosiglitazone alone could effectively induce a brown-like phenotype in mouse iWAT-SVCs, and this had been well documented in previous reports [4,8]. However, rosiglitazone and CL316,243 mediated distinct sets of brown genes (Figure 4A). Rosiglitazone is superior at inducing higher levels of mitochondrial function genes such as *Cidea*, *Cox5b*, *Cox7a1*, *Cox8b*, and *Cycs*, while CL316,243 is more potent to induce the brown marker genes, including *Ucp1*, *Pgc1α*, and *Dio2*. Notably, white adipocyte-selective genes, *Leptin* and *Resistin* are more responsive to rosiglitazone than CL316,243. Our finding suggested that CL316,243 and rosiglitazone have unique responding pathways and downstream targets during adipocyte browning. It is reasonable that CL316,243 promote browning through the β3-adrenergic pathway, while rosiglitazone induce brown genes and repress white genes by activation of PPARγ and stabilization of PRDM16 protein [16,17].

It had been proposed that RG-induced brite adipocytes were different from CL-induced beige adipocytes [10]. Beige adipocytes had been found to arise from progenitors expressing TMEM26 or CD137 as well as smooth muscle-like markers [16,18]. However, the precise origin of the brite adipocytes is still not known. To date, it seems brite cells only arise from unnatural stimulus such as rosiglitazone or roscovitine, unlike the physiological case with cold induction of beige cells [10]. Shingo and colleagues reported that combination therapy with capsinoids and mild cold exposure synergistically promotes adipocyte browning and prevents diet-induced obesity [19]. They also verified that capsinoids promote browning by stimulating a stabilization of the PRDM16 protein [19]. Intriguingly, PPARγ agonists rosiglitazone induce a white-to-brown fat conversion also through stabilization of PRDM16 protein [17]. Thus, we speculate that combining β3 adrenergic pathway and PRDM16 pathway would be an effective strategy to counteract obesity.

However, our results demonstrated that CL316,243 and rosiglitazone could additively but not synergistically induce thermogenic gene expression (Figure 4A,B). Even though there were no expected synergies, the combination of these two drugs still had many advantages. Firstly, *Ucp1*, the hallmark gene of browning that produces heat by uncoupling electron transport from ATP synthesis during adaptive thermogenesis, significantly increased because of the combination of CL316,243 and rosiglitazone, compared with treatment with either CL316,243 or rosiglitazone alone. Secondly, the combination of CL316,243 and rosiglitazone immensely increased the expression of genes involved in mitochondrial biogenesis, such as *Cidea*, *Cox5b*, *Cox7a1*, *Cox8b*, and *Cycs*. Mitochondrial biogenesis is an important step during the activation of thermogenesis [20]. A similar effect was reported in brown adipocyte thermogenesis by combination of rosiglitazone and forskolin/norepinephrine [21,22]. Thus, it might be a promising way to develop a therapy strategy to induce browning through combining use CL316,243 and rosiglitazone.

As depicted in Figure 5A, it seems rosiglitazone and CL316,243 preferentially activates different metabolic pathways involved in adipocyte browning. For instance, CL316,243 mainly promotes fat acid β-oxidation, while rosiglitazone causes an increase of cAMP biosynthesis; both metabolic pathways are important mediators of adipocyte browning [23]. Thus, we speculated that the additive effect of promoting thermogenic gene expression of CL316,243 and rosiglitazone is partly due to their complementary role on metabolic pathways activation.

Furthermore, the combination of CL316,243 and rosiglitazone improved the status of insulin sensitivity by downregulating *Resistin* expression but without affecting *Adiponectin* expression (Figure 4D). *Resistin* and *Adiponectin* are two important adipokines secreted from white adipocytes, especially mature white adipocytes, and are known to be implicated in insulin resistance [24,25]. A significantly negative correlation between *Resistin* levels and insulin sensitivity was previous observed in other reports, while *Adiponectin* levels showed a positive correlation with insulin sensitivity [26]. In our current study, we found a significant downregulation of *Adiponectin* after CL316,243 stimuli, co-treatment with rosiglitazone was able to reverse the upregulation of *Resistin*, and downregulation of *Adiponectin* resulted from CL316,243 stimuli. Thus, besides the additive effect of promoting thermogenic gene expression and mitochondrial biogenesis, it is reasonable to combine these two agents to eliminate the side effect on insulin sensitivity.

## 4. Materials and Methods

### 4.1. Isolation of Adipose SVF and In Vitro Differentiation

Animal experiments were performed according procedures approved by the Institutional Animal Care and Use Committees of Northwest A&F University. SVF cells isolated by collagenase digestion of minced mice inguinal WAT were plated onto collagen-coated dishes and cultured in 5% CO_2_ at 37 °C. WAT SVF cells were expanded in growth media containing DMEM/F12 (Invitrogen, Carlsbad, CA, USA) and 10% fetal bovine serum (FBS). For white adipocyte differentiation, confluent cultures were stimulated with an adipogenic cocktail (growth media supplemented with 5 μg·mL^−1^ insulin, 1 μM dexamethasone, 0.5·mM isobutylmethyxanthine, and 1·μM rosiglitazone) for 48 h. After 48 h, the cells were maintained in growth media supplemented with 5 μg·mL^−1^ insulin until harvest.

The cells were treated with CL316,243 (2 µM, Tocris Bioscience, Bristol, UK) 4 h before harvest. After CL316,243 treatment, mature adipocytes were lysed in Trizol (TaKaRa, Naha, Japan) for RNA extraction.

### 4.2. Oil Red O staining

Cells were fixed with 10% formalin and incubated for 30 min at room temperature with gentle shaking. The cells were then washed with 60% isopropanol and incubated in Oil Red O working solution. After 10 min of staining, the plates were rinsed with distilled water four times.

### 4.3. Gene Expression Analysis

Total RNA was extracted from cultured cells by using RNAiso Plus Reagent (TaKaRa) and a synthesized First Strand cDNA with PrimeScript^®^ RT Reagent Kit (TaKaRa). The sequences of the primers used in this study are listed in Table S1. Relative expression of mRNAs was determined by qPCR using the SYBR Green PCR system (Applied Biosystems, Foster City, CA, USA), and values were normalized to β-actin. The 2^−△△*C*t^ method was chosen to analysis the data (△△*C*t = △*C*t − △*C*t).

### 4.4. Protein Analysis

Total protein from cultured cells was harvested in an extraction buffer (Beyotime, Shanghai, China) supplemented with protease and phosphatase inhibitors (Pierce, Rockford, IL, USA). Briefly, protein samples were separated using 10% SDS-PAGE gels, then transferred to polyvinylidene difluoride membranes (Millipore, Bedford, MA, USA). Membranes were blocked with 5% nonfat dry milk in TBS containing 0.1% Tween for 1 h at room temperature and then blotted with primary antibodies: anti-UCP1 (1:1000), anti-β-tubulin (1:1000), anti-FABP4 (1:1000), and anti-PGC1α (1:1000) overnight. After washing, membranes were incubated with a secondary horseradish peroxidase (HRP)-coupled antibody and visualized using Immobilon HRP substrate (Millipore, Billerica, MA, USA). The density of the bands was quantified using ImageJ Software (National Institute of Health, Bethesda, MD, USA). The ratio of the intensity of the target protein to that of β-tubulin loading control was calculated to represent the expression level of the protein.

### 4.5. Immunofluorescence

Cells were fixed with formaldehyde for 10 min, then washed with PBS and penetrated with 0.1% Triton X-100. Samples were incubated in blocking buffer containing 3% BSA for 1 h at room temperature. Samples were then incubated with UCP1 antibody at a 1:200 dilution for 2 h. Cells were incubated with red fluor secondary antibody for 1.5 h at 37 °C and nuclei were stained with DAPI (Sigma, St. Louis, MO, USA).

### 4.6. Bioinformatic Analysis

Microarray data (GSE87,191: the mRNA signature of white adipocytes, brite adipocytes, and beige adipocytes) was obtained from the GEO (Gene Expression Omnibus) database using GEO2R (an online tool to identify genes that are differentially expressed across different experimental conditions) for screening differentially expressed genes, and the Biocyc database (BioCyc integrates sequenced genomes with predicted metabolic pathways for thousands of organisms and provides extensive bioinformatics tools) was used to perform pathway enrichment analysis for these differentially expressed genes.

### 4.7. Data Analysis

Statistical analysis was performed using SPSS13.0. Comparisons between the two groups were analyzed by paired Student’s *t*-tests. Comparisons among groups were made by one-way ANOVA test. Data are presented as mean ± SEM of independent experiments. *p* < 0.05 was set as statistical significance.

## 5. Conclusions

In summary, our study highlighted the significance of the additive effect of promoting thermogenic gene expression by a combination of PPARγ pathway and β3-Adrenergic pathway, which might be useful in developing a valid therapeutic implication for anti-obesity.

## Supplementary Materials

Supplementary materials can be found at www.mdpi.com/1422-0067/18/5/1002/s1.

## Abbreviations

iWAT-SVCs	Inguinal white adipose tissue derived stromal vascular cells
eWAT	Epididymal white adipose tissue
*Ppar* *α/γ*	Peroxisome proliferator activated receptor alpha/gamma
*Fabp4*	Fatty acid binding protein 4
*Ucp1*	Uncoupling protein 1
*Pgc1α*	Peroxisome proliferative activated receptor, gamma, coactivator 1 alpha
*Cidea*	Cell death-inducing DNA fragmentation factor, alpha subunit-like effector A
*Dio2*	Deiodinase, iodothyronine, type II
*Retn*	Resistin
*Prdm16*	PR domain containing 16
*Zfp423*	Zinc finger protein 423
*Ebf2*	Early B cell factor 2
*Cycs*	Cytochrome c, somatic
*Cox5b/7a1/8b*	Cytochrome c oxidase subunit 5b/7a1/8b
*Adrb1*	Adrenergic β1 receptor
*Tnf (ip3/6)*	Tumor necrosis factor (induced protein 3/6)
*Elovl3*	Elongation of very long chain fatty acids
*Slc25a20/27a2*	Solute carrier family 25 member 20/ Solute carrier family 27 member 2
*Tlr2/7*	Toll-like receptor 2/7
*Gpr126*	G protein-coupled receptor 126
*Tmem43*	Transmembrane protein 43
