# Supplementary material for: An Additive Effect of Promoting Thermogenic Gene Expression in Mice Adipose-Derived Stromal Vascular Cells by Combination of Rosiglitazone and CL316,243
Source: Int J Mol Sci. 2017 May 8;18(5):1002. doi: 10.3390/ijms18051002 (PMC5454915; doi:10.3390/ijms18051002)
Supplement: Supplementary file 1 [file ijms-18-01002-s001.zip › Table S1- qPCR primers used in this study.pdf]

Table S1: Primers used for qPCR.

Gene	Forward primer (5' - 3')	Reverse primer (5' - 3')
**common adipocyte genes**		
AdipoQ	GCACTGGCAAGTTCTACTGCAA	GTAGGTGAAGAGAACGGCCTTGT
Pparγ	GTGCCAGTTTCGATCCGTAGA	GGCCAGCATCGTGTAGATGA
Fabp4	ACACCGAGATTTCCTTCAAACTG	CCATCTAGGGTTATGATGCTCTTCA
**thermogenic genes**		
Prdm16	CAGCACGGTGAAGCCATTC	GCGTGCATCCGCTTGTG
Ebf2	GCTGCGGGAACCGGAACGAGA	ACACGACCTGGAACCGCCTCA
Cidea	TGCTCTTCTGTATCGCCCAGT	GCCGTGTTAAGGAATCTGCTG
Pgc1α	CCCTGCCATTGTTAAGACC	TGCTGCTGTTCCTGTTTTC
Ucp1	ACTGCCACACCTCCAGTCATT	CTTTGCCTCACTCAGGATTGG
Dio2	CAGTGTGGTGCACGTCTCCAATC	TGAACCAAAGTTGACCACCAG
Cox5b	GCTGCATCTGTGAAGAGGACAAC	CAGCTTGTAATGGGTTCCACAGT
Cox7a1	CAGCGTCATGGTCAGTCTGT	AGAAAACCGTGTGGCAGAGA
Cox8b	GAACCATGAAGCCAACGACT	GCGAAGTTCACAGTGGTTCC
Cycs	GCAAGCATAAGACTGGACCAAA	TTGTTGGCATCTGTGTAAGAGAATC
**beige adipocyte selective genes**		
CD137	CGTGCAGAACTCCTGTGATAAC	GTCCACCTATGCTGGAGAAGG
Tmem26	ACCCTGTCATCCCACAGAG	TGTTTGGTGGAGTCCTAAGGTC
**white-selective genes**		
Retn	CTGTCCAGTCTATCCTTGCACAC	CAGAAGGCACAGCAGTCTTGA
Leptin	GAGACCCCTGTGTCGGTTC	CTGCGTGTGTGAAATGTCATTG
**reference gene**		
β-actin	CGTGAAAAGATGACCCAGATCA	CACAGCCTGGATGGCTACGT
